# Spatial Representation of Hippocampal Place Cells in a T-Maze with an Aversive Stimulation

**DOI:** 10.3389/fncir.2017.00101

**Published:** 2017-12-11

**Authors:** Sakura Okada, Hideyoshi Igata, Takuya Sasaki, Yuji Ikegaya

**Affiliations:** ^1^Graduate School of Pharmaceutical Sciences, University of Tokyo, Tokyo, Japan; ^2^Center for Information and Neural Networks, Suita, Japan

**Keywords:** hippocampus, place cell, aversive location, learning, remapping

## Abstract

The hippocampus contains place cells representing spaces in an environment, and these place cells have been suggested to play a fundamental role in the formation of a cognitive map for spatial processing. However, how alterations in the firing patterns of place cells in response to aversive events encode the locations tied to these aversive events is unknown. Here, we analyzed spiking patterns of place cell ensembles in the dorsal hippocampal CA1 region of rats performing a T-maze alternation task with an aversive air-puff stimulation applied at a specific location on one side of a trajectory. The intensity of the air puff was adjusted so that the rats decreased their running speed before passing the aversive location. The addition of the aversive stimulus induced reorganization of place cell ensembles on both left and right trajectories with and without the aversive stimulus, respectively. Specifically, the animals showed a more abundant spatial representation in the vicinity of the aversive location. Removing the aversive stimulus induced new spatial firing patterns on both of the trajectories that differed from those both before and during application of the aversive stimulus. These results demonstrate that hippocampal spatial maps are flexibly reorganized to represent particular aversive events.

## Introduction

In natural environments, animals are surrounded by aversive stimuli that generate negative emotional valence. As aversive stimuli are linked with particular places, animals need to learn the association between a space and an aversive event and decide whether to initiate avoidance behavior against aversive locations. In some circumstances, they need to accept aversive situations to obtain an anticipated reward to ensure their survival.

A possible neural substrate underlying spatial memory and spatial navigation is hippocampal place cells, which specifically fire at local regions of an environment (O’Keefe and Dostrovsky, 1971) and are assumed to constitute a cognitive map in the brain (O’Keefe and Nadel, 1978). Introducing an aversive stimulus into a space has been demonstrated to alter the spatial representation of place cells (Moita et al., 2004; Oler et al., 2008; Wang et al., 2012; Kim et al., 2015; Wu et al., 2017), including substantial shifts in place fields and the development of new place fields. The flexible reorganization of hippocampal cell spatial firing patterns might be a neural mechanism for learning novel aversive events in a given environment and navigational behavior decision-making.

Although early studies tested how the addition of aversive events affects place cell firing, a technical limitation in these studies was that they utilized electrical shocks, causing a large electrical noise artifact in the electrophysiological recording system, making it impossible to monitor neuronal activity patterns during the electrical shock (Moita et al., 2003, 2004; Wu et al., 2017). While some studies have reported alterations of place cell firing without electrical shocks by utilizing a looming robot (Kim et al., 2015) or predator odor (Wang et al., 2012), these studies were not designed to record neuronal activity in a specific region where the aversive stimulus was presented. Consequently, how hippocampal place cells represent a fixed location tied to an aversive event remains to be directly tested. Addressing this question would reveal how hippocampal neurons incorporate information regarding aversive events into an existing spatial framework and, furthermore, how the hippocampal place cells are involved in spatial navigation including the decision of whether to take approach/avoidance behavior against aversive events.

To describe neuronal firing patterns in an aversive location, two experimental conditions are required: (1) recording data must not be contaminated by electrical noise, and (2) animals need to repeatedly enter an aversive location. In this study, we designed a novel spatial task that incorporates these technical requirements by modifying a conventional T-maze alternation task (Wood et al., 2000; Ito et al., 2015). Here, rats were exposed to an aversive air-puff stimulation before reaching an area associated with a reward. Utilizing air puff as an aversive stimulus allowed us to record spike patterns of multiple hippocampal neurons without suffering from electrical noise. The intensity of the air-puff stimulation was adjusted so that the rats showed behavioral signs of hesitation before entering the aversive location. Using this experimental paradigm, we analyzed how spatial representations of place cell ensembles in the dorsal CA1 region are altered by a newly emerged aversive situation.

## Materials and Methods

### Approvals

This study was carried out in accordance with the recommendations of the NIH guidelines for the care and use of animals. The protocol was approved by the experimental animal ethics committee at the University of Tokyo (approval number: P29-7).

### Subjects

A total of 7 male Long Evans rats (5–11 months old) with a preoperative weight of 300–450 g were used in this study. The animals were housed individually and maintained on a 12-h light/12-h dark schedule with lights off at 7:00 AM. All animals were purchased from SLC (Shizuoka, Japan). Following at least 1 week of adaptation to the laboratory, the rats were reduced to 85% of their ad libitum weight through limited daily feeding. Water was readily available.

### Apparatus and Behavioral Task

**FIGURE 1:**
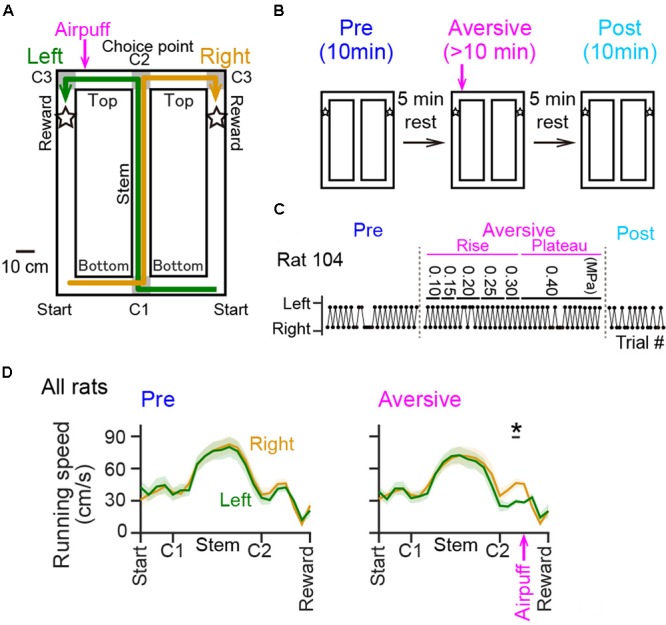
Behavioral performance of rats in a T-maze alternation task with an air-puff stimulation. **(A)** An overview of the T-maze. Noteworthy locations are labeled Start, Bottom (between Start and C1) C1, Stem (between C1 and C2), C2 (choice point), Top (between C2 and C3), C3, and Reward (indicated by stars). The location of the air-puff stimulation is indicated by the magenta arrow. **(B)** A recording day was composed of a series including a 10-min pre-session, a >10-min aversive session during which an air-puff stimulation was introduced at the aversive location (magenta arrow), and a 10-min post-session with an intersession interval of 5 min. **(C)** Behavioral results of left-right choices observed from a rat. The values shown above represent the intensity of the air puff (in MPa) applied during the aversive session. **(D)** Running speed averaged over all rats was plotted against the position in the pre-session [Pre; *F*(1,252) = 0.01, *P* = 0.90, repeated measures ANOVA] and in the plateau phase of the aversive session [Aversive; *F*(1,252) = 0.29, *P* = 0.59, repeated measures ANOVA]. The top black bar indicated by the asterisk represents that a significant difference was observed between the left and right trials. The magenta arrow indicates the location of the air-puff stimulation. ^∗^*P* < 0.05, paired *t*-test.

The T-maze used in this study was 90 cm × 120 cm, 74 cm elevated from the floor, and made of ABS resin. The maze consisted of the central runway (stem) and two lateral runways with a length of 120 cm (**Figure 1A**) for returning from reward areas to the next start points. All alleyways had a width of 10 cm and were surrounded by a transverse wall with a height of 5 cm. The maze was placed on an experimental room with a size of 150 cm × 200 cm and with a height of 280 cm. Single room cues with a shape of star, circle, and square with a size of ∼30 cm, were attached to individual room walls at a height of 150 cm. The top side of the maze was partially open to an experimenter and the recording device. The animals could view all of these room cues and the experimenter elsewhere on the maze. As shown in **Figure 1A**, noteworthy locations were labeled as start, corner1 (C1), Stem (between C1 and C2), corner 2 (C2; choice point), Top (between C2 and C3), corner 3 (C3), and reward areas (areas 10 cm after both sides of C3, indicated by stars). Green and yellow arrows in **Figure 1A** show correct left-right trajectory patterns, respectively. The experimenter provided 0.2 ml of chocolate milk on the reward area.

In addition to the T-maze apparatus, a custom-made photosensor to detect animal’s crossing was attached to the wall 20 cm after the choice point on the top alleyway specifically on the left trajectory, and an airpuff generator to apply airpuff to the animals was attached to the wall 5 cm after the photosensor (termed as the aversive location). The timing of airpuff application was regulated by opening a solenoid valve.

### Training of the Task

Before surgery, the rats were trained to perform a continuous spatial alternation task on the T-maze. The rules of the task is similar to those in previous studies (Wood et al., 2000; Ito et al., 2015). All behavioral experiments occurred in the dark phase. In the training phase, the airpuff generator was turned off. On the first 2–3 days, the rats were habituated to the T-maze by allowing them to freely forage for randomly scattered chocolate milk for 10 min. After the habituation period, the animals started a training phase of the T-maze task.

In each time of taking a trajectory, the experimenter provided a chocolate milk reward on either one of reward areas. The animals were trained to follow a direction on the maze, from one side of a start point, the Stem area, the opposite side of the Top area and a reward area, to acquire a reward. After taking a reward, the animals were instructed to go down the lateral alley and cross the opposite side of the start point. A next reward was provided on the opposite side of the reward area when the animals’ head entered the stem. The animals could obtain the next reward if they could choose the opposite trajectory, compared with the preceding trial. During the training days, reverse movement was blocked by the experimenter’s hands or a transparent plastic barrier so that the animals could learn to alternately take left-right trajectories and run in a “figure 8”-like pattern. In each day of the training phase, this procedure was repeated as much as possible in a 10-min session. The training was repeated until the animal was able to consume reward at least 20 times within the 10-min session per day. To reach this criterion, the training lasted 10–14 days. The rats were kept on a rest box (33 × 33 cm) outside the field for tens of minutes before and after the training.

### Surgical Procedures

After reaching the criterion in the training phase, the rat was anesthetized with isoflurane gas (2–3%). A craniotomy of a diameter of ∼2 mm was performed using a high-speed drill, and the dura was surgically removed. Two stainless-steel screws were implanted in the bone above the frontal cortex to serve as ground and reference electrodes. An electrode assembly that consisted of 8–16 independently movable tetrodes, which was created using a 3-D printer (MiiCraft+, Young Optics), was stereotaxically implanted above the right hippocampus (4.0 mm posterior and 2.7 mm lateral to bregma). The tip of the electrode bundle was lowered to the cortical surface, and the electrodes were inserted 1.0 mm into the brain at the end of surgery. The electrodes were constructed from 17-μm-wide polyimide-coated platinum-iridium (90/10%) wire (California Fine Wire), and the electrode tips were plated with platinum to lower electrode impedances to 150–300 kΩ at 1 kHz. The recording device was secured to the skull using stainless steel screws and dental cement. Following surgery, each rat was housed individually in transparent Plexiglass with free access to water and food for at least 3 days. After recovery form surgery, food was deprived to 85% of their body weight.

### Adjusting Electrode Depth

The rat was connected to the recording equipment via Cereplex M (Blackrock), a digitally programmable amplifier, close to the rat’s head. The output of the headstage was conducted via a lightweight multiwire tether and a commutator to the Cerebus recording system (Blackrock), a data acquisition system. The depth of electrodes was adjusted while the rat was resting on a pot placed on a pedestal. Over a period of at least 1 week after surgery, electrode tips were advanced slowly 25–250 μm per day until spiking cells were encountered in the CA1 layer of the hippocampus, which was identified on the basis of local field potential (LFP) signals and single-unit spike patterns. Once the tetrodes were adjacent to the cell layer, as indicated by the presence of multiunit activity, tetrodes were settled into the cell layer for stable recordings over a period of several days.

During this period of adjusting depth of electrodes, a behavioral training of the same T-maze alternation task was resumed 7.2 ± 2.9 days (ranging from 4 to 11 days) after surgery. This post-surgery training lasted for at least 2 days before performing electrophysiological recording. In some cases, the training was performed with the recording headstage and cable attached to the animal’s head so that the animal got familiar with the recording condition.

### Electrophysiological Recording

Electrophysiological data recording began after the animals again reached the criterion same as in the training phase and stable well-separated unit activity was identified in the hippocampus. In a recording day, the animals performed a series of three task sessions; (1) a 10-min pre-session, (2) a >10-min aversive session, and (3) a 10-min post-session. After performing each session, the animal was allowed to rest for 5 min during which the floor of the field was cleaned with water and 70% ethanol. The behavioral paradigm of the pre-session was completely identical to that of the a 10-min session of the training phase. In the aversive session, the airpuff generator was turned on and an airpuff stimulation was transiently (∼500 ms) applied to the animals in the aversive location, which was triggered by the photosensor that detects crossing of the animals on left trajectories. No stimulation was applied when the animals took the opposite right trajectories. The pressure of the aversive airpuff stimulation was manually adjusted by an experimenter using a pressure regulator, ranging from 0.01 to 0.45 MPa, so that the animals showed reduced running speed before entering the aversive point on left trajectories but kept the motivation to actively traverse the aversive point. In this condition, no intense behavioral signs such as jumping and freezing were observed. In the post-session, the airpuff stimulation was removed so that experimental conditions are similar to those of the pre-session.

Local field potential recordings were sampled at 2 kHz and filtered between 0.1 and 500 Hz. Unit activity was amplified and band-pass filtered at 500 to 6 kHz. Spike waveforms above a trigger threshold (40 μV) were time-stamped and recorded at 30 kHz for 1.6 ms. To monitor the rat’s moment-to-moment position, a red LED was attached to the animal’s back and the position of the LED signal was tracked at 25 Hz using a video camera attached to the ceiling, which was sampled by a laptop computer.

### Histological Analysis to Confirm Electrode Locations

The rats received an overdose of sodium pentobarbital and were perfused intracardially with 4% paraformaldehyde in phosphate buffered saline (pH 7.4) and decapitated. To aid the reconstruction of electrode tracks, the electrodes were not withdrawn from the brain until 3–4 h after perfusion. After dissection, the brains were fixed overnight in 4% PFA and then equilibrated with a sequence of 20% sucrose and 30% sucrose in phosphate-buffered saline. Frozen coronal sections (40 μm) were cut using a microtome, and serial sections were mounted and processed for cresyl violet staining. For cresyl violet staining, the slices were rinsed in water, counterstained with cresyl violet, and coverslipped with Permount. The positions of all tetrodes were confirmed by identifying the corresponding electrode tracks in histological tissue. Recordings were included in the data analysis if the tetrode’s deepest position was in the cell layer.

### Spike Sorting

Spike sorting was performed offline using the graphical cluster-cutting software MClust (Redish, 2009). Sleep recordings before and after the behavioral paradigms were included in the analysis to assure recording stability throughout the experiment and to identify hippocampal cells that were silent during behavior. Clustering was performed manually in 2D projections of the multidimensional parameter space (i.e., comparisons between waveform amplitudes, the peak to trough amplitude differences, and waveform energies, each measured on the four channels of each tetrode). Only clusters that could be stably tracked across all behavioral sessions were considered to be the same cells and were included in our analysis. Auto-correlation and cross-correlation functions were used as additional separation criteria. Cells with an average firing rate of less than 6 Hz and waveforms longer than 200 μs were considered to be putative excitatory cells and included in analysis.

### Analysis of Behavioral Patterns

A period during taking left or right trajectories was termed as left or right trials, respectively. An incorrect trial was defined if the animals entered the opposite side of a correct trajectory. In the next trial, a correct trajectory was defined depending on the start point at which the animals passed. For quantification, the animal’s path was linearized by projecting the animal’s coordinate onto a center line of alleyways corresponding with each trajectory. An instantaneous speed at each frame was calculated based on the total distance traveled within a period five frames (∼200 ms) before and after the focused frame.

### Spatial Firing Patterns of Individual Neurons

**FIGURE 2:**
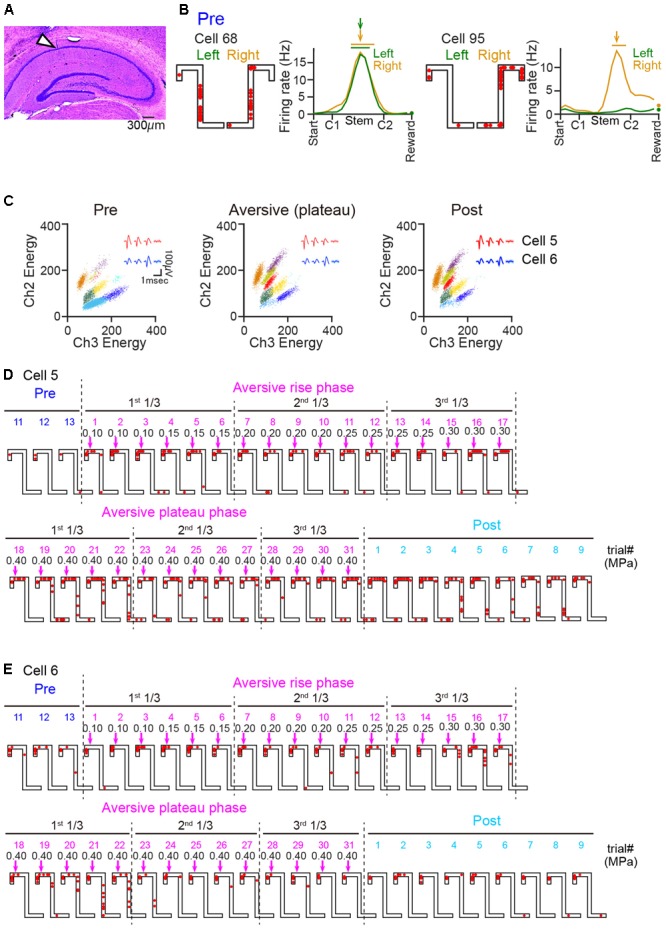
Changes in the spatial firing of hippocampal place cells after the introduction of an air-puff stimulation. **(A)** Histological confirmation of a recording site in the dorsal hippocampal CA1 cell layer in a cresyl-stained section. The arrowhead indicates the track of an electrode. **(B)** Spatial firing patterns of two representative CA1 cells in the pre-session. Cell 68 shows place-selective firing at the same location in both trajectories, whereas cell 95 shows spatial selectivity specifically in the right trajectory. For each cell, the left panel shows spike locations in a left and right trial of the pre-session as indicated by the red dots superimposed on the maze structure, and the right panel shows the firing-rate distribution on the left and right trajectories averaged over the pre-session. The total number of spikes in a given location bin was normalized by the total time spent in that location. The place field center is indicated by the colored arrows, and the range of the place field is indicated by the horizontal bars above the graph. Firing rates at bins including reward were separately plotted as dots because the duration spent in the reward bins was considerably different from that spent elsewhere in the maze. **(C)** Cluster plots showing the energy of multiunit signals recorded from two channels. Each dot represents one spike and each color represents each cluster that was assigned to a single cell. The insets in individual sessions represent average spike waveforms of cell 5 (red) and cell 6 (blue). The spatial firing patterns of these cells are shown in **D** and **E**. **(D,E)** Spatial firing patterns of two representative cells in consecutive left trials including the last three trials in the pre-session and all trials in the aversive session. The colored values above the maze represent trial numbers in each session. The magenta arrows and the black numbers indicate the position and the intensity (in MPa) of the air-puff stimulation, respectively. In the aversive session, the trials were classified into a rise phase and plateau phase, and each phase was further divided into three periods (1st, 2nd, and 3rd 1/3-periods).

For analyzing spike patterns, the positions of spikes of individual cells were projected onto a center line of alleyways corresponding with each trajectory same as the animal’s coordinate. Average firing-rate distribution on each correct left-right trajectory was separately computed along the projected line by dividing the total number of spikes in each location bin (10 cm) by the total time that the animal spent in that bin. The location bins of the reward area were excluded from subsequent analysis as the duration the animal spent in these areas was considerably different from those elsewhere on the maze. All firing-rate distributions were smoothed by a one-dimensional convolution with a Gaussian kernel with a standard deviation of one pixel (10 cm). Based on an average spatial firing-rate distribution, a place filed in a left or right trajectory in one session was defined with two following criteria: (1) a firing-rate distribution in a session had the maximum firing rate of more than 3 Hz, and (2) the maximum firing rate exceeded 2 standard deviations (SDs) above the mean, where the SD and the mean were computed from the series of firing rates except the maximum firing rate in that distribution. The second criterion computes spatial selectivity within the firing-rate distribution. The place field center was defined as the position giving the maximum firing rate in the distribution. The range of a place field was defined by iteratively extending the field from the place field center to any adjacent bins that had firing rates of >30% of the maximum rate (an example shown in **Figure 2A**). Under this criterion, some place cells had one place field in either one of two trajectories whereas the others had two place fields in both of trajectories. In the following analyses, place fields from a single place cell were counted as two place fields and were separately analyzed.

**FIGURE 3:**
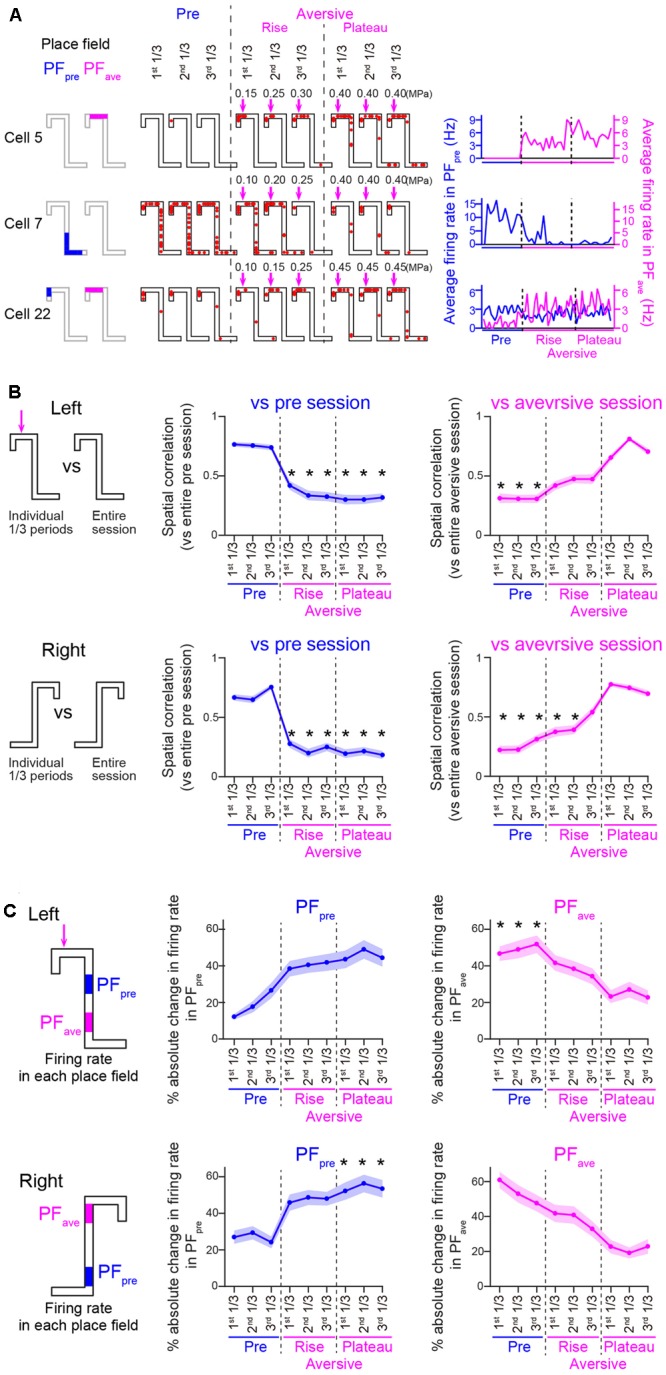
**(A)** Spatial firing patterns of three representative cells on left trials. (Left) Place fields identified in the pre- and aversive sessions, termed PF_pre_ and PF_ave_, are shown by colored regions on the maze. (Middle) Typical spatial firing for a single left trial during three individual periods of the pre-session and the phases of the aversive session. (Right) Changes in average firing rates computed within the PF_pre_ and PF_ave_ for individual left trials. **(B)** Plots of spatial correlations of averaged firing-rate distributions between individual period and entire pre-session (left; *n* = 45 and 69 cells in Left and Right trials, respectively) or entire plateau phase of the aversive session (right; *n* = 45 and 51 cells in Left and Right trials, respectively). Left and right trajectories are separately shown. Asterisks indicate significant differences between all three periods of the pre-session (left panels) or all three periods of the plateau phase of the aversive session (right panels), by Kruskal–Wallis test with Tukey–Kramer *post hoc* test. ^∗^*P* < 0.05. **(C)** Same as in **C** but plotted for absolute change ratios of average firing rates in the PF_pre_ (left; *n* = 45 and 69 cells in Left and Right trials, respectively) and PF_ave_ (right; *n* = 45 and 44 cells in Left and Right trials, respectively). In each place field, the ratio of the average firing rate in each period to the average firing rate in the pre-session or the plateau phase of the aversive session was computed for the PF_pre_ or PF_ave_, respectively. Asterisks are the same as in **B**.

For each animal, the aversive session was divided into two phases: (1) a rise phase during which the intensity of airpuff stimulation was increased before reaching the maximum, and (2) a plateau phase during which the stimulus intensity was maintained constant at its maximum. The pre-session, the individual phases in the aversive session, the post-session, were each divided into three periods with equivalent numbers of trials: first 1/3, second 1/3, and third 1/3 periods (**Figures 3, 7**). Place fields identified in the pre-session, the plateau phase of the aversive session, and the post-session, were termed as PF_pre_, PF_ave_, and PF_post_, respectively. Within each place field on a left trajectory, firing rates were computed every trial (**Figures 3A, 7A**). In each period *i*, an absolute change ratio of a firing rate within a place field was computed as abs[(FR_i_–FR_ref_)]/(FR_i_+FR_ref_), where FR_i_ and FR_ref_ were an average firing rate within the place field in the period *i* and an average firing rate within the place field that was calculated from the entire session defining the place field, respectively (**Figures 3C, 7C**).

### Statistics

All data are presented as mean ± standard error of the mean (SEM) and were analyzed using Python and Matlab. Comparison of spatial distributions of place field centers between left and right trajectories were assessed using Kolmogorov–Smirnov test. Multiple group comparisons of spatial distributions of running speed were assessed using a repeated measures ANOVA. Multiple group comparisons of absolute change ratios of firing rates were assessed using Kruskal–Wallis test with Tukey–Kramer *post hoc* test. Comparison of ratio distributions of place field types between two groups were assessed using Chi-square test. The null hypothesis was rejected at the *P* < 0.05 level, unless otherwise specified.

## Results

### Behavioral Performance in a T-Maze Alternation Task with an Aversive Stimulus

Rats were well-trained to perform a continuous T-maze alternation task in which they took left and right trajectories on alternating laps to obtain a reward placed at fixed locations (**Figure 1A**). In a recording day, the rats performed a series of three sessions, a 10-min pre-session, a >10-min aversive session, and a 10-min post-session (**Figure 1B**). In the pre-session, the average percentages of correct trials in all seven animals were 96.1 ± 2.1% and 88.0 ± 3.6% for the left and right trajectories, respectively, and were not different between the two trajectory types [**Figure 1C**; *t*(6) = 2.13, *P* = 0.077, paired *t*-test]. In addition, no differences were found in the spatial distribution of running speed between the left and right trajectories in all animals tested [**Figure 1D** left, *F*(1,252) = 0.01, *P* = 0.90, repeated measures ANOVA; data from individual animals are shown in **Table 3**], confirming that the animals took both trajectories equivalently with similar running patterns in the pre-session. In the next aversive session, an air-puff stimulation was applied to the rats when they traversed the aversive location, which was 25 cm after the choice point in the left trajectory, indicated by the magenta arrow in **Figure 1A**. For the animal shown in **Figure 1**, the stimulus intensity was first set at 0.10 MPa, the minimum intensity, and was gradually increased to 0.40 MPa, the maximum intensity. The maximum intensity was determined by observing each animal’s behavioral response to the stimulation and varied across animals (**Table 1**). In the period with the maximum intensity, termed the plateau phase of the aversive session, the proportion of correct trials over the number of total trials using the left trajectory was not prominently different from that using the right trajectory [**Table 1**; *t*(6) = 1.96, *P* = 0.097, paired *t*-test], but the average running speed in the 10-cm bin 10 cm after C2, the choice point, in the left trials, corresponding with the 10-cm bin 5 cm before the aversive location, was significantly lower than that in the right trials [**Figure 1D** right and **Table 2**; *t*(6) = 3.64, *P* = 0.0011, paired *t*-test]. This result demonstrates that rats showed a behavioral sign of hesitation when passing the aversive location.

**Table 1:** Summary of behavioral performance in all animals.

Rat #	Session	% Correct trials		Airpuff intensity (MPa)
		Left	Right	
Rat 68	Pre-session	25/27 (92.6%)	29/29 (100%)	–
	Aversive session (plateau phase)	19/20 (95%)	21/21 (100%)	0.45
	Post-session	16/17 (94.1%)	15/16 (93.8%)	–
Rat 104	Pre-session	15/15 (100%)	14/17 (82.4%)	–
	Aversive session (plateau phase)	15/18 (83.3%)	14/14 (100%)	0.4
	Post-session	13/14 (92.9%)	9/9 (100%)	–
Rat 105	Pre-session	18/20 (90%)	17/21 (81.0%)	–
	Aversive session (plateau phase)	5/6 (83.3%)	4/4 (100%)	0.08
	Post-session	8/10 (80%)	8/8 (100%)	–
Rat 140	Pre-session	15/15 (100%)	14/17 (82.4%)	–
	Aversive session (plateau phase)	8/14 (57.1%)	9/10 (90%)	0.02
	Post-session	3/3 (100%)	3/3 (100%)	–
Rat 165	Pre-session	33/33 (100%)	32/32 (100%)	–
	Aversive session (plateau phase)	26/31 (83.9%)	26/26 (100%)	0.45
Rat 167	Pre-session	9/10 (90%)	7/8 (88%)	–
	Aversive session (plateau phase)	8/9 (88.9%)	9/9 (100%)	0.15
	Post-session	9/9 (100%)	9/9 (100%)	–
Rat 195	Pre-session	6/6 (100%)	5/6 (83%)	–
	Aversive session (plateau phase)	6/6 (100%)	9/11 (82%)	0.15
	Post-session	2/2 (100%)	4/4 (100%)	–

*The percentages of correct trials were calculated as the number of correct trials divided by the number of total trials. Airpuff intensity at the plateau phase in the aversive session is shown at the right column.*

**Table 2:** Summary of running speed at specific areas of all animals.

Rat #	Session	Speed at stem (cm/s)		Speed at the area 10 cm after C2		
				(In the left trajectory, the area is 5 cm before the aversive location)		
		Left	Right	Left	Right	Left vs. Right
Rat 68	Pre-session	81.7 ± 5.1	79.4 ± 11.5	55.9 ± 4.3	49.1 ± 3.9	*t*(40) = -1.76, *P* = 0.08
	Aversive session (plateau phase)	76.4 ± 5.9	79.7 ± 8.6	30.5 ± 1.8 (vs. pre, *t*(38) = 5.50, ^∗^*P* = 2.76E-06)	46.3 ± 3.8	*t*(64) = 3.06, ^∗^*P* = 0.003
	Post-session	68.8 ± 7.0	77.7 ± 9.1	48.0 ± 6.5 (vs. aversive, *t*(32) = -3.02, ^∗^*P* = 0.005)	30.4 ± 4.8	*t*(24) = -0.92, *P* = 0.37
Rat 104	Pre-session	60.3 ± 7.4	58.8 ± 11.4	42.2 ± 3.5	52.4 ± 6.5	*t*(27) = 2.83, ^∗^*P* = 0.009
	Aversive session (plateau phase)	41.0 ± 10.1	51.6 ± 7.2	30.1 ± 1.5 (vs. pre, *t*(25) = 3.23, ^∗^*P* = 0.003)	37.1 ± 5.2	*t*(26) = 4.03, ^∗^*P* = 0.0004
	Post-session	37.1 ± 7.1	44.1 ± 7.4	25.6 ± 1.9 (vs. aversive, *t*(21) = 1.85, *P* = 0.47)	37.1 ± 1.5	*t*(19) = 6.15, ^∗^*P* = 6.52E-06
Rat 105	Pre-session	53.5 ± 12.4	54.3 ± 7.7	43.1 ± 3.2	47.6 ± 3.1	*t*(32) = -0.16, *P* = 0.88
	Aversive session (plateau phase)	47.4 ± 4.8	54.0 ± 6.8	23.9 ± 2.8 (vs. pre, *t*(34) = 4.46, ^∗^*P* = 0.0001)	50.2 ± 2.1	*t*(35) = 2.64, ^∗^*P* = 0.012
	Post-session	37.2 ± 7.7	45.0 ± 8.5	43.1 ± 1.4 (vs. aversive, *t*(24) = -4.34, ^∗^*P* = 0.0002)	42.9 ± 2.8	*t*(14) = -0.74, *P* = 0.47
Rat 140	Pre-session	48.5 ± 12.6	53.2 ± 14.4	36.1 ± 3.3	40.3 ± 5.1	*t*(25) = 3.37, ^∗^*P* = 0.024
	Aversive session (plateau phase)	25.7 ± 17.9	31.9 ± 19.0	24.9 ± 5.9 (vs. pre, *t*(20) = 2.27, ^∗^*P* = 0.035)	34.7 ± 7.4	*t*(14) = 1.56, *P* = 0.14
	Post-session	23.2 ± 8.6	13.8 ± 5.1	31.1 ± 8.3 (vs. aversive, *t*(8) = -0.89, *P* = 0.40)	20.2 ± 6.8	*t*(3) = -0.62, *P* = 0.58
Rat 165	Pre-session	68.7 ± 9.2	77.2 ± 4.1	55.5 ± 0.7	65.4 ± 2.6	*t*(63) = 9.14, ^∗^*P* = 3.65E-13
	Aversive session (plateau phase)	57.5 ± 6.0	69.9 ± 10.6	29.3 ± 0.9 (vs. pre, *t*(86) = 19.57, ^∗^*P* = 1.99E-33)	51.0 ± 2.4	*t*(107) = 13.67, ^∗^*P* = 3.17E-25
Rat 167	Pre-session	26.1 ± 9.5	23.2 ± 8.3	35.9 ± 2.7	41.0 ± 2.5	*t*(7) = 0.79, *P* = 0.45
	Aversive session (plateau phase)	25.5 ± 10.8	23.2 ± 13.6	31.0 ± 3.5 (vs. pre, *t*(12) = 1.16, *P* = 0.27)	46.6 ± 9.7	*t*(20) = 1.34, *P* = 0.20
	Post-session	33.8 ± 19.9	31.9 ± 15.8	61.6 ± 10.5 (vs. aversive, *t*(12) = -3.17, ^∗^*P* = 0.008)	48.3 ± 4.7	*t*(12) = 1.77, *P* = 0.10
Rat 195	Pre-session	16.2 ± 7.8	19.9 ± 11.0	33.7 ± 8.1	31.8 ± 6.4	*t*(9) = -0.39, *P* = 0.70
	Aversive session (plateau phase)	21.0 ± 9.0	23.5 ± 8.5	29.1 ± 5.2 (vs. pre, *t*(13) = -0.75, *P* = 0.47)	26.8 ± 5.3	*t*(13) = -0.75, *P* = 0.47
	Post-session	13.0 ± 4.1	11.4 ± 6.5	16.7 ± 5.7 (vs. aversive, *t*(10) = 1.28, *P* = 0.23)	23.8 ± 6.7	*t*(4) = 1.05, ^∗^*P* = 0.35

*(Left two columns) Average running speed at stem. (Right three columns) Average running speed at the 10-cm area 10 cm after C2. In the left trajectory, the area corresponds to the 10-cm area 5 cm before the aversive location. The rightmost column shows statistical results of the comparison of the speed between left and right trials.*

**Table 3:** (Left two columns) The statistical results of the comparison of speed spatial distributions among the pre, aversive and post-sessions computed by repeated measures ANOVA.

Rat #	Session	Comparison of speed distribution among pre, aversive, and post-session		Comparison of speed distribution between left and right trials
		Left	Right	
Rat 68	Pre-session	*F*(2,1071) = 2.28, *P* = 0.1127	*F*(2,1617) = 2.88, *P* = 0.0624	*F*(1,840) = 3.48, *P* = 0.070
	Aversive session (plateau phase)			*F*(1,1344) = 0.16, *P* = 0.6894
	Post-session			*F*(1,504) = 0.50, *P* = 0.485
Rat 104	Pre-session	*F*(2,693) = 58.13, *P* = 0	*F*(2,819) = 24.83, *P* = 0	*F*(1,567) = 1.40, *P* = 0.25
	Aversive session (plateau phase)			*F*(1,546) = 9.52, ^∗^*P* = 0.0048
	Post-session			*F*(1,399) = 13.85, ^∗^*P* = 0.0014
Rat 105	Pre-session	*F*(2,861) = 8.66, *P* = 0.0007	*F*(2,840) = 6.53, *P* = 0.0035	*F*(1,672) = 0.35, *P* = 0.56
	Aversive session (plateau phase)			*F*(1,735) = 5.50, ^∗^*P* = 0.024
	Post-session			*F*(1,294) = 4.10, *P* = 0.062
Rat 140	Pre-session	*F*(2,441) = 9.36, *P* = 0.012	*F*(2,441) = 13.41, *P* = 0.0002	*F*(1,525) = 1.83, *P* = 0.19
	Aversive session (plateau phase)			*F*(1,294) = 0.01, *P* = 0.92
	Post-session			*F*(1,63) = 0.72, *P* = 0.46
Rat 165	Pre-session	*F*(2,1911) = 29.79, *P* = 0	*F*(2,1911) = 71.32, *P* = 0	*F*(1,1323) = 2.90, *P* = 0.092
	Aversive session (plateau phase)			*F*(1,2247) = 13.94, ^∗^*P* = 0.0003
Rat 167	Pre-session	*F*(2,357) = 4.25, *P* = 0.0318	*F*(2,462) = 0.33, *P* = 0.7192	*F*(1,147) = 0.31, *P* = 0.59
	Aversive session (plateau phase)			*F*(1,420) = 0.43, *P* = 0.52
	Post-session			*F*(1,252) = 0.68, *P* = 0.43
Rat 195	Pre-session	*F*(2,231) = 1.67, *P* = 0.2322	*F*(2,315) = 0.17, *P* = 0.17	*F*(1,147) = 0.31, *P* = 0.59
	Aversive session (plateau phase)			*F*(1,420) = 0.43, *P* = 0.52
	Post-session			*F*(1,252) = 0.68, *P* = 0.43

*(Right column) The statistical results of the comparison of speed spatial distributions between the left and right trajectories in individual sessions computed by repeated measures ANOVA.*

### Changes in Spatial Firing Induced by the Aversive Stimulus

A total of 146 cells were analyzed and 102 hippocampal neurons in the dorsal hippocampal CA1 region were identified from 7 rats during the task sessions (**Figure 2A**). **Figure 2B** shows average spatial firing-rate distributions of two example cells in the pre-session. In the pre-session, 49 cells had place fields on both trajectories, and 19 and 19 cells had place fields on either the left or right trajectory, respectively, resulting in a total of 136 identified place fields. All subsequent analyses were restricted to these place cells. Out of the 68 and 68 place fields on the left and right trajectories, 33 (48.5%) and 32 (47.1%) fields were located on the Stem area, respectively, and 23 (33.8%) and 22 (32.4%) fields were located on the Top area, respectively. No significant difference in the spatial distributions of the place field centers was found between the left and right trajectories (*D*_max_ = 0.14, *P* = 0.98, Kolmogorov–Smirnov test), verifying that both left and right trajectories were equivalently represented in the pre-session.

Next, the firing patterns in the aversive session were analyzed. The total numbers of trials in the aversive session were 92 trials (Rat 68), 64 trials (Rat 104), 36 trials (Rat 105), 16 trials (Rat 140), 109 trials (Rat 165), 19 trials (Rat 167), and 28 trials (Rat 195). We first confirmed the stability of spike waveforms across the three sessions. **Figure 2C** shows representative projection patterns of the energy of spikes recorded from a single tetrode. The figure demonstrates that the patterns of these spike clusters (labeled with each color) remained stable over the three sessions. Likewise, all spike clusters identified from the other tetrodes had stable spike waveforms, verifying that increased and decreased number of spikes recorded across the sessions was not due to the cells having moved in or out of the spike waveforms. The spatial firing patterns of these two representative cells identified in **Figure 2C** in consecutive left trials are shown in **Figures 2D,E**. In each animal, three periods, that is the first 1/3, second 1/3, and third 1/3-periods, with equivalent numbers of left trials were defined in each phase of the aversive session, the rise phase and plateau phase. Cell 5 fired very few spikes in the pre-session and then started to fire when the aversive session started (**Figure 2D**). Cell 6 showed robust spatial firing in all the recording sessions (**Figure 2E**). Detailed characteristics of these spatial firing patterns were quantified in the following analyses.

**Figure 3A** shows the spatial firing patterns of three representative cells. Place fields identified in the pre-session and the plateau phase of the aversive session were termed PF_pre_ and PF_ave_, respectively. Conceptually, firing patterns of PF_pre_ represent the spatial firing pattern observed before adding the aversive stimulus and were maintained across the following sessions, whereas those in PF_ave_ represent newly emerged spatial firing patterns induced by the aversive stimulus and were maintained across the sessions. Cell 5 began to fire in its PF_ave_ immediately after the aversive stimulus was applied; cell 7 showed stable firing rates in its PF_pre_ in the pre-session but eliminated its firing field gradually in the aversive session; and cell 22 initially had a place field near the reward location and extended its firing field toward C2 including the aversive location after the aversive stimulus emerged, implying that this cell might be responsible for a prospective coding of the incoming aversive stimulus.

To assess changes in spatial firing patterns, spatial correlation analysis was applied (**Figure 3B**). **Figure 3B** left panels show spatial correlations of averaged firing-rate distribution between individual 1/3 periods and an entire pre-session. On both trajectories, no significant differences in the spatial correlations were found in all 1/3 periods of the pre-session whereas correlations were significantly decreased in all periods of the aversive session [**Figure 3B**, left; ^∗^*P* < 0.05, Tukey–Kramer *post hoc* test versus all 1/3 periods of the pre-session; Left trials: *F*(8,396) = 122.9, *P* = 8.4 × 10^-23^; Right trials: *F*(8,612) = 217.2, *P* = 1.5 × 10^-42^, Kruskal–Wallis test]. Notably, the spatial correlations on both of the two trajectories were significantly different from those in any periods of the rise phases (**Figure 3B**, left; *P* > 0.05, Tukey–Kramer *post hoc* test), suggesting that spatial firing patterns became similar to those observed in the plateau phase immediately after the addition of the aversive stimulus. **Figure 3B** right panels show spatial correlations of averaged firing-rate distribution between the individual 1/3 periods and an entire plateau phase of the aversive session. On both trajectories, the correlations were significantly lower in the pre-session [**Figure 3B**, right; ^∗^*P* < 0.05, Tukey–Kramer *post hoc* test versus all 1/3 periods of the plateau phase of the aversive session; Left trials: *F*(8,296) = 103.1, *P* = 9.8 × 10^-19^; Right trials: *F*(8,450) = 142.6, *P* = 6.7 × 10^-27^, Kruskal–Wallis test]. These results demonstrate that spatial firing patterns in the aversive session were prominently different from those observed in the pre-session and emerged immediately after learning the presence of aversive stimulus.

**FIGURE 4:**
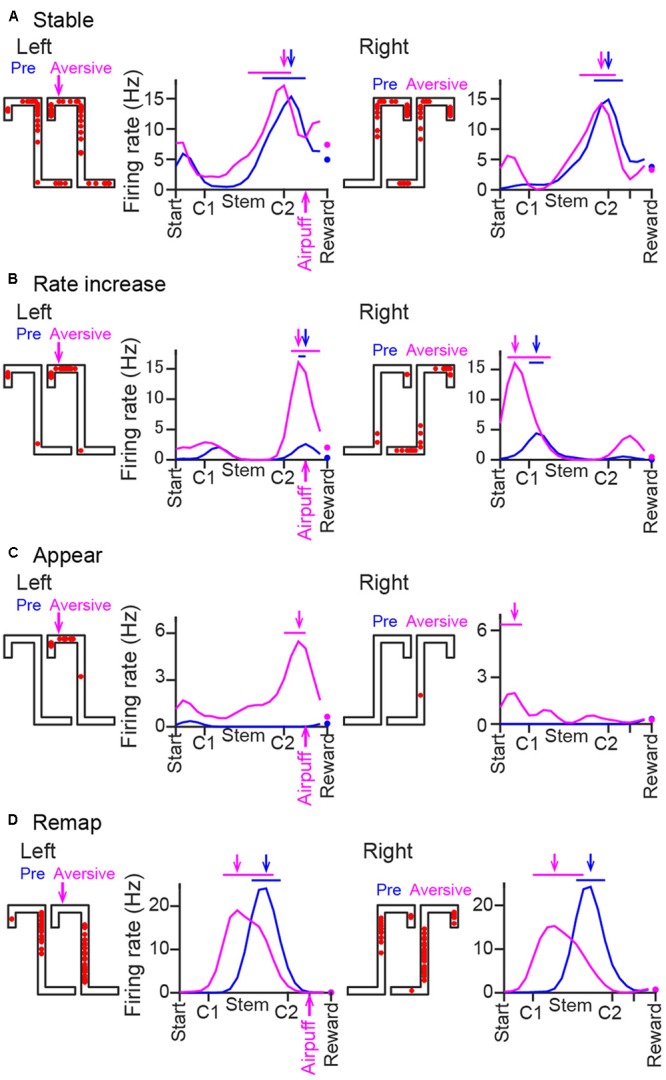
Changes in the spatial firing patterns of representative cells. **(A–D)** Spike locations and average firing-rate distribution of three representative place cells, shown the same as in **Figure 2B** except that the left and right trajectories were separately plotted with data on the same trajectory obtained from the pre-session (blue) and the plateau phase of the aversive session (magenta) superimposed. These examples include a cell with no pronounced changes in spatial firing patterns (**A**, stable), a cell with an increase in a firing rate at the aversive location (**B**, rate increase), a cell with a new place field at the aversive location (**C**, appear), and a cell with shifting of its place fields (**D**, remap) across the two sessions.

To further confirm this result, absolute change ratios of average firing rates within the PF_pre_ and PF_ave_ on the left and fight trajectories in individual cells were also computed (**Figure 3C**). In the aversive session, the absolute change ratios within the PF_pre_ showed a significant change across periods [**Figure 3C**, left; Left trials: *F*(8,441) = 57.2, *P* = 1.7 × 10^-9^; Right trials: *F*(8,486) = 53.6, *P* = 8.2 × 10^-9^, Kruskal–Wallis test]. Absolute change ratios of firing rates computed within the PF_ave_ on the left trajectory in the plateau phase of the aversive session were significantly different from those of the pre-session [**Figure 3C**, right; ^∗^*P* < 0.05, Tukey–Kramer *post hoc* test, between all periods of the plateau phase of the aversive session and all periods of the pre-session; Left trials: *F*(8,441) = 57.2, *P* = 1.7 × 10^-9^; Right trials: *F*(8,486) = 53.6, *P* = 8.2 × 10^-9^, Kruskal–Wallis test]. The observation that change ratios observed in the plateau phase of the aversive session were lowered to approximately 10%, suggested that firing patterns in the plateau phase were almost stabilized after introducing the aversive stimulus. Based on this assumption, these firing patterns were analyzed as representative firing patterns in the aversive session in the classification analyses from **Figure 4**.

### Spatial Firing in Incorrect Trials

**FIGURE 5:**
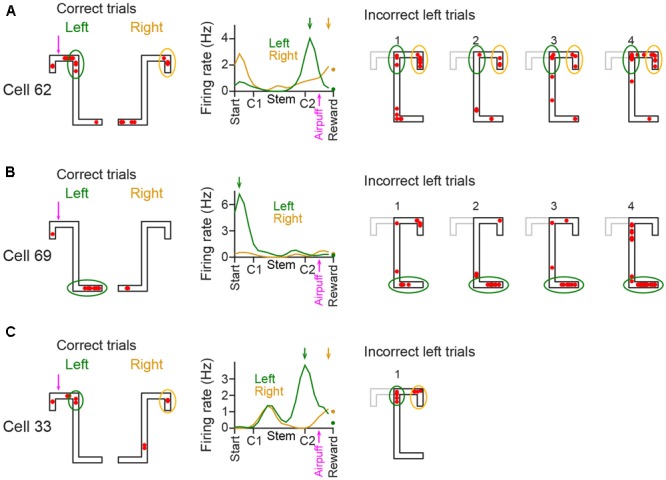
Spatial firing in incorrect trials in the plateau phase of the aversive session. (**A–C**; Left three panels) Spatial firing patterns of representative CA1 cells in correct trials in the plateau phase of the aversive session. The data are shown same as in **Figure 2B**. In the spatial map, typical areas with increased firing rates are enclosed by colored circles (left trials, green; right trials, yellow). (Right panels) Spatial firing patterns of the same cells observed in individual incorrect trials. The areas corresponding with the left panels are enclosed by colored circles, same as in the left panels. The correct left trajectory patterns are depicted in the gray lines.

We analyzed whether spatial firing patterns varied between correct and incorrect behavioral performance (**Figure 5**). In each place cell, the area where its spatial firing rate was increased until reaching C2 in incorrect left trials seemed similar to that in correct left trials as indicated by the green circles. In addition, the area showing increased firing rates after passing C2 in incorrect left trials seemed similar to that in correct right trials as indicated by the yellow circles. These results imply that spatial firing patterns in incorrect behavior include the information of both left and right trajectories and emerged in reference to animal’s absolute positions. Detailed analyses based on spatial firing-rate distribution were not performed due to the limited number of incorrect trials.

### Classification of Change Patterns of Spatial Firing Induced by the Aversive Stimulus

Spatial firing patterns were compared between the pre-session and the plateau phase of the aversive session (**Figure 5**). The emergence of a new aversive stimulus induced diverse types of changes in place cell populations. **Figure 4A** shows a place cell with no pronounced changes in spatial firing rates across sessions. Here, such place fields that shifted their field center with a distance of less than 30 cm across sessions without changing the in-field firing rate were classified as “stable.” **Figure 4B** shows a place cell that initially had a place field located before crossing the aversive point in the pre-session and further increased its firing rate without changing the position of the place field in the presence of the air-puff stimulation. Here, such place fields that increased the in-field firing rate by more than two times were classified as “rate increase.” **Figure 4C** shows a place cell that was initially silent in the pre-session but newly generated a place field in the aversive session, classified as “appear.” In contrast, place cells that were initially apparent in the pre-session but eliminated their place fields in the aversive session were classified as “disappear.” **Figure 4D** shows cells that shifted their place fields to a new location in the aversive session, classified as “remap.”

**FIGURE 6:**
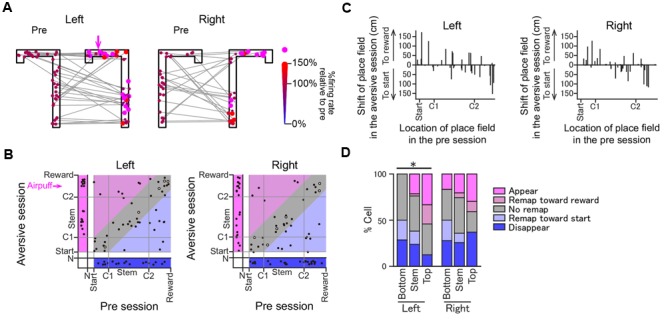
Changes in the positions of place fields induced by the introduction of the aversive stimulus. **(A)** Positions of the place field centers of all place fields from the pre-session to the plateau phase of the aversive session are indicated by colored dots. For visualization purposes, the positions of the dots were randomly jittered less than 5 cm in the space. Shifts of the place field centers across the two sessions are indicated by gray lines. In each place field, the dot in the aversive session is plotted in a different size and color depending on the ratio of the firing rate at the place field center in the aversive session to that in the pre-session. Place fields that newly emerged in the aversive session are represented by magenta dots. **(B)** In each place field, the position of the place field center in the plateau phase of the aversive session was plotted against that in the pre-session. Areas under start, indicated by N, represent cells that exhibited no spatial firing in one session but spatial firing in the other session. Types of changes in spatial firing patterns are shown in colored area as follows: magenta, appear; light magenta, remap toward reward; gray, stable; light blue, remap toward start; blue, disappear. The open dots represent cells showing >2× increases in the maximum firing rates in the aversive session. **(C)** The shifts of place fields from the pre-session to the aversive session plotted against the locations of place field centers in the pre-session. **(D)** The proportions of place fields classified into the types shown in **B**. Data were separately analyzed for the Bottom, Stem, and Top areas (^∗^*P* < 0.05, Chi-square test with Bonferroni correction).

The positions of all place field centers observed in the two sessions are shown in **Figure 6A**. Out of the 69 and 64 fields identified in the left and right trajectories in the aversive session, 19 (27.5%) and 23 (35.9%) fields newly appeared in the aversive session, respectively, plotted in the magenta area in **Figure 6B**. Out of the 69 and 64 fields identified in the left and right trajectories in the pre-session, 18 (26.1%) and 27 (42.2%) fields disappeared in the aversive session, respectively, plotted in the blue area. Out of the 33 and 27 place fields that did not show a shift of the place field center, plotted in the gray area, 28 (40.6%) and 21 (32.8%) fields were classified as “stable” in the left and right trajectories, respectively. **Figure 6C** summarizes the shift of their place fields from the pre-session to the aversive session. The proportions of these cell types were further assessed by separating trajectories into the Top, Stem, and Bottom areas (**Figure 6D**). Out of all fields identified, the percentage of new fields in the Top area in the left trajectory was 33.3%, which was higher than that in the Stem area (21.4%) and the Bottom area (0%) in the left trajectory and those in the right trajectory. In contrast, the percentage of fields that disappeared in the Top area in the left trajectory was 12.5%, which was lower than that in the Stem and Bottom area in the left trajectory (23.8 and 28.6%, respectively) and those in the right trajectory. The differences between the ratio distributions in the left trajectories, but not in the right trajectories, shown in **Figure 6D** (Left trials, χ^2^ = 23.7, *P* = 0.044; Right trials, χ^2^ = 12.1, *P* = 0.88 chi-square test with Bonferroni corrections) were statistically significant. Overall, the results indicate that adding an aversive stimulus led to a higher probability of spatial representation on the left trajectories at the vicinity of the aversive location (Top area) than elsewhere in the maze. We need to note that this analysis did not take running speed-dependent increases in firing rates of place cells into consideration (McNaughton et al., 1983; Ahmed and Mehta, 2012; Zheng et al., 2015). Therefore, the number of cells showing firing rate increases might be overestimated at the area where the running speed was increased (e.g., the middle of the stem as shown in **Figure 1D**). Nonetheless, we found a higher probability of spatial representation of place cells before the aversive location where running speed was lowered, which suggests that the place-selective firing at this area is not simply explained by changes in running speed.

### Change Patterns of Spatial Firing after the Removal of the Aversive Stimulus

**FIGURE 7:**
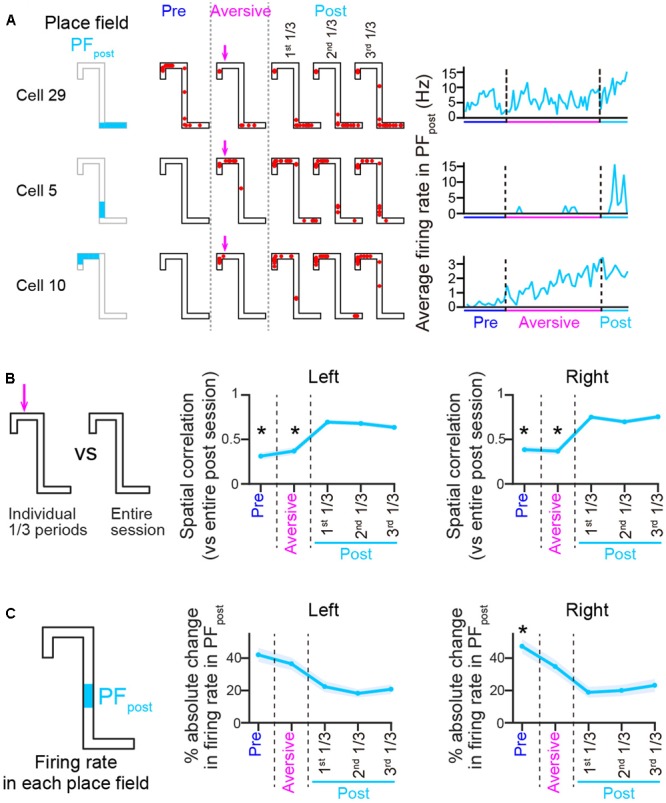
Spatial firing patterns in the post-session differ from those both in the pre- and aversive sessions. **(A)** Spatial firing patterns of three representative cells in the post-session, shown the same as in **Figure 3A**. Place fields identified in the post-sessions were termed PF_post_. **(B)** Plots of spatial correlations of averaged firing-rate distributions between entire pre-session, entire aversive session, and individual 1/3 periods of the post-session and entire post-session, shown the same as in **Figure 3B** (*n* = 50 and 69 cells in Left and Right trials, respectively). The asterisks indicate significant differences between all three periods of the post-session, by Kruskal–Wallis test with Tukey–Kramer *post hoc* test. ^∗^*P* < 0.05. **(C)** Plots of absolute change ratios of average firing rates in the PF_post_, shown the same as in **Figure 3C** (*n* = 50 and 59 cells in Left and Right trials, respectively). Asterisks are the same as in **B**.

Finally, we tested how spatial firing was altered after removing the aversive stimulus in the post-session (*n* = 6 animals). The percentage of correct left trials [**Table 1**; *t*(5) = 1.41, *P* = 0.22] was not significantly different from those in the aversive session. In three out of six animals tested, the running speed before entering the aversive location in the left trajectory in the post-session was significantly higher than that in the aversive session (**Table 2**), showing that some animals could partially restore their behavioral patterns in the post-session as the rats learned that the aversive stimulus was no longer present. **Figure 7A** shows the change patterns of spatial firing of three example cells from the pre-session, the aversive session, and the post-session. **Figure 7B** left panel shows spatial correlations of averaged firing-rate distribution between an entire pre-session, entire post-session, and individual 1/3 periods of the post-session and an entire post-session. On both trajectories, the correlations were significantly lower in the pre and aversive session [**Figure 7B**, left; ^∗^*P* < 0.05, Tukey–Kramer *post hoc* test versus all 1/3 periods of the post-session; Left trials: *F*(4,245) = 48.6, *P* = 7.1 × 10^-10^; Right trials: *F*(4,340) = 82.1, *P* = 6.3 × 10^-17^, Kruskal–Wallis test]. Same as in **Figure 3C**, absolute change ratios of firing rates in place fields identified in individual periods were also computed relative to an average firing rate in the PF_post_ over the course of the entire post-session, showing that the absolute change ratios showed a significant change across periods [**Figure 7C**; ^∗^*P* < 0.05, Tukey–Kramer *post hoc* test versus all 1/3 periods of the post-session; Left trials: *F*(4,190) = 19.7, *P* = 6.0 × 10^-4^; Right trials: *F*(4,290) = 37.4, *P* = 1.5 × 10^-7^, Kruskal–Wallis test]. These results suggest that the firing patterns induced by removing the aversive stimulus in the post-session differed from those observed in the pre and aversive sessions.

## Discussion

In this study, we designed a new behavioral paradigm by incorporating a modest air-puff stimulation into a T-maze alternation task. In this task condition, the rats exhibited a slower running speed, especially before crossing the aversive point, representing a behavioral sign of hesitation, but were still motivated to run across the aversive location to obtain an expected reward. This behavior allowed us to record how hippocampal neurons of the dorsal hippocampal CA1 region encode the location associated with an aversive stimulus. Our analysis of neuronal firing patterns revealed that (1) the emergence of an aversive stimulus induced new spatial representations on both trajectories, (2) changes in the new spatial representation included both increases in in-field firing rates of place cells and the appearance of new place fields, especially in the vicinity of the aversive location, and (3) removal of the aversive stimulus in the post-session induced new firing patterns that were different from those observed both before introducing the aversive stimulus in the pre-session and in the presence of the aversive stimulus. A limitation of our task design was that the aversive location was close to the rewarded goal location. Therefore, we note a possibility that firing patterns observed at the vicinity of aversive area might be a mixed representation of the aversive event and prospective coding of a reward location.

Previous reports have shown that the addition of a goal such as a reward area or escape platform in a new location within a maze induces an accumulation of place fields at the goal location (Hollup et al., 2001; Kobayashi et al., 2003; Dupret et al., 2010) and higher firing rates (Hok et al., 2007; McKenzie et al., 2013) near the goal location, suggesting that hippocampal place cells over-represent locations that generate positive emotional valence. These changes in firing patterns are consistent with our observations of the emergence of new place fields near a new aversive location. Taken together, increased spatial representation is a shared neurophysiological basis for hippocampal neurons to encode the locations of behavioral significance, irrespective of valence type. Furthermore, increases in the number of place fields were observed in the Top area, including the regions preceding the aversive location on the left trajectory, suggesting that spatial representation in the Top area might include prospective information, which might be used to predict near future aversive events. Consistent with this observation, spatially extended prospective firing of place cells has been observed at a location preceding a rewarding goal (Ferbinteanu and Shapiro, 2003; Hok et al., 2007; Catanese et al., 2014; Wikenheiser and Redish, 2015). Taken together, enhanced spatial representation of future locations is a shared neurophysiological basis for hippocampal neurons to encode the future events of behavioral significance.

While there are some similarities between the representation patterns of hippocampal place cells in response to rewarding goals and aversive events, different neuronal mechanisms might be used to create these plastic changes in the excitability of hippocampal neurons. Our finding that some neurons rapidly showed a new spatial representation within a few trials after the addition of an aversive stimulus is unlikely to be explained by a long-term stress-induced endocrine response or change in gene expression. An alternative plausible mechanism is that plastic changes occur in the dynamic interactions between the hippocampus and the amygdala, which is considered to be a key neuronal mechanism to associate spatial, contextual information with emotional, aversive information. In line with this hypothesis, recent reports have demonstrated that place cell activity is modulated by the manipulation of amygdala activity. First, disruption of amygdalar signaling influences the stability of hippocampal place cells in a fear environment (Donzis et al., 2013; Kim et al., 2015). Second, amygdalar activation causes reorganization of hippocampal place cell maps (Kim et al., 2012). The evidence supports the idea that the abundant spatial representation near the aversive locations found in our study also depends on the amygdala fear system.

Our data showed that the firing patterns after the removal of the aversive stimulus in the post-session differed from those observed in both the pre-session and the aversive session, demonstrating that they do not revert to previous patterns exhibited before learning. This finding is consistent with those observed in the other studies (Tronson et al., 2009; Grewe et al., 2017) and supports an idea that memory extinction is not a true elimination of learned aversive memory but is a formation of a new memory that aversive events are no longer present in the environment (Amano et al., 2010; Duvarci and Pare, 2014).

We note that the pronounced reorganization of place fields was also observed on the Stem and Bottom areas on the left trajectory and the entire areas on the right trajectory in which no aversive event occurred. This finding implies that an aversive experience at a specific location can affect the spatial representation throughout the entire maze. Other possible explanations are that this reorganization might be caused by the appearance of novelty or changes in animal’s attention and motivational states outside the aversive location. Further studies with more appropriate task designs are required to separately analyze the effects of these individual factors on the learning-induced spatial representation.

Our findings add to an accumulating body of evidence of the nature of the hippocampal signals related to aversive behavior and spatial representation. The over-representation of aversive locations by place cells may contribute to the assimilation of new information into the existing spatial map, and the potentiated place fields may serve as a memory index within the cognitive map. As the firing patterns of these place fields associated with an aversive stimulus showed some similarities with reward-induced place cell signals (Hollup et al., 2001; Kobayashi et al., 2003; Dupret et al., 2010), an unresolved question is how the increased firing rates of hippocampal place cells in response to both positive (reward) and negative (aversive) valence are distinguished by downstream brain circuits, leading to opposite behavioral patterns, such as approach/avoidance behavior. Future studies using a combination of multi-neuron electrophysiology and genetically targeted technologies are required to unveil how the information flow is distributed across multiple brain circuits.

## Author Contributions

SO and TS designed the work. SO and HI acquired electrophysiological data. SO and TS performed analysis. YI supervised the project. SO and TS prepared all figures. TS wrote the main manuscript text and all authors reviewed the main manuscript text.

## Conflict of Interest Statement

The authors declare that the research was conducted in the absence of any commercial or financial relationships that could be construed as a potential conflict of interest.
